# Recurrent TET2 mutations in adult T cell leukemia

**DOI:** 10.1186/1742-4690-11-S1-P119

**Published:** 2014-01-07

**Authors:** Ambroise Marçais, Katia Hanssens, Lucy Cook, Thomas Mercher, Philippe Gaulard, Vahid Asnafi, Claudine Pique, Ali Bazarbachi, Felipe Suarez, Olivier Bernard, Charles RM Bangham, Patrice Dubreuil, Olivier Hermine

**Affiliations:** 1 Service d’Hématologie, Hôpital Universitaire Necker, Université René Descartes, Institut Imagine, Paris, France; 2 Inserm U1068, Centre de Recherche en Cancérologie de Marseille, Institut Paoli-Calmettes, Université de la Méditerranée, Marseille, France; 3 Department of Immunology, Wright-Fleming Institute, Imperial College, London, UK; 4 Inserm U985, Institut Gustave Roussy, Université Paris-Sud 11, Villejuif, France; 5 Département de Pathologie, Groupe Henri Mondor Albert Chenevier, Créteil, France; 6 Department d’Hématologie, Hôpital Universitaire Necker, Université René Descartes, Paris, France; 7 Inserm, U1016, Institut Cochin, Paris, France; 8 Department of Internal Medicine, American University of Beirut, Beirut, Lebanon

## 

Deregulation of DNA methylation has been recently identified in malignant hematologic diseases such as inactivation of the Ten-ElevenTranslocation 2 (TET2) gene by haplo-insufficiency. Inactivating mutations of TET2 were first described in myeloid disorders and more recently in peripheral T-cell lymphomas especially those that are harboring T follicular helper features like angio-immunoblastic T cell lymphoma. In order to determine new oncogenic pathways in Adult T cell leukemia/lymphoma (ATLL) that could cooperate with viral oncogenic proteins, we investigated the presence of TET2 coding sequence mutations and their clinical relevance in a retrospective cohort of 49 ATL patients. We identify inactivating mutations of TET2 gene in 10 patients of 49 analyzed (20%). Of the 37 patients with aggressive forms (acute and lymphoma), 9 (24%) had TET2 mutations as only one (8%) of the 12 with indolent forms had a TET2 mutation. This last patient had nevertheless a poor outcome and died four years from the diagnosis from relapse. In addition, five patients show the same recurrent point mutation, which conducts to the loss of coding sequence in one allele and lead to haplo-insufficiency. Analysis of different clinical cases suggests that TET2 mutation could be acquired at different steps of the T cell oncogenesis and could even in some cases precede HTLV-1 infection. Analysis of viral integration is still ongoing. Here, we show that loss of TET2 is frequently associated with ATLL and seems to more frequent in aggressive forms.

